# Repeatability of a multi-segment foot model with 15-marker set in normal adults

**DOI:** 10.1186/1757-1146-7-S1-A74

**Published:** 2014-04-08

**Authors:** Sang Gyo Seo, Dong Yeon Lee, Ji-Beom Kim, Seong Hyun Kim, Hye Sun Park, Hyo Jeong Yoo, Sung Ju Kim, Jihyeung Kim, Kyoung Min Lee, Chin Youb Chung, In Ho Choi

**Affiliations:** 1 Department of Orthopedic Surgery, Seoul National University Hospital, Seoul, Korea; 2 Department of Statistics, Korea University, Seoul, Korea; 3 Department of Orthopedic Surgery, Seoul National University Boramae Medical Center, Seoul, Korea; 4 Department of Orthopedic Surgery, Seoul National University Bundang Hospital, Seongnam, Korea

## 

Several 3D multi-segment foot models (MFMs) have been introduced for the in vivo analysis of dynamic foot kinematics [1,2]. However, there is scanty evidence available to support their clinical use. Considering the potential of MFM to assess the function in foot pathology, there is a need for simple, reproducible and reliable multi-segment foot models. The purpose of this study was to assess the reliability of a simple MFM with 15-marker set.

Twenty healthy adults mean aged 28.9 years (10 males and 10 females) were tested. Eight markers of 15-marker set were placed in foot to evaluate segmental foot motion. Three representative strides from five separate trials were used for analysis from each session. Kinematic data of foot segmental motion was collected and tracked using the Foot3D Multi-Segment Software (Motion Analysis Co., Santa Rosa. CA). Retests were performed in the same manner with an interval of 4 weeks. Coefficients of multiple correlation (CMC) and intra-class correlation (ICC) were calculated in order to assess the inter-trial and inter-session repeatability. Inter-segment foot angles from healthy adults from a MFM with 15-marker set showed a narrow range of variability during the whole gait cycle.

**Figure 1:**
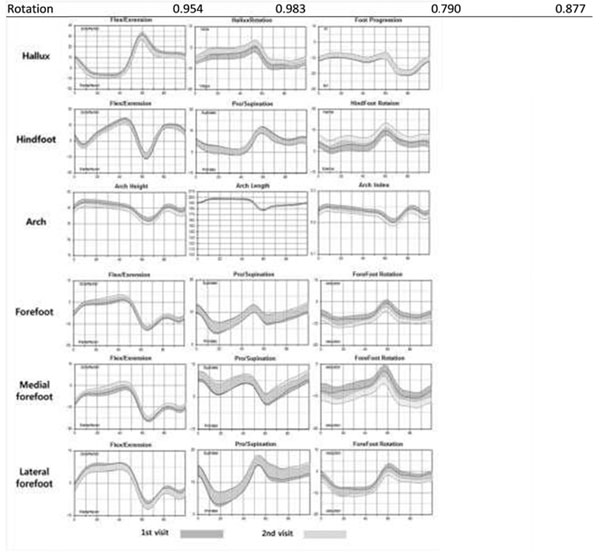
Walking kinematics for the 1st and 2nd visit (average with a range representing 2 standard deviations). Each row shows the motion of each segment: hallux, hindfoot, arch, forefoot, medial forefoot, lateral forefoot motion. Each column represents motion in each of the three planes (sagittal, coronal, transverse plane). Horizontal axis represents gait cycle, and vertical axis represents range of motion.

The mean inter-trial ICC (± Standard deviation) was 0.981 (± 0.010), which was interpreted as excellent. The mean inter-trial CMC (± Standard deviation ) was 0.948 (± 0.027), which was interpreted as excellent or very good repeatability. The mean inter-session ICC (±SD) was 0.886 (± 0.047) and the mean inter-session CMC (±SD) was 0.801 (± 0.077), which were interpreted as excellent or very good repeatability. The lowest repeatability was in the transverse plane at the forefoot and the most consistent finding was observed at the sagittal plane of the hallux and hindfoot (Table 1, Figure 1).

**Table 1:** Repeatability of foot kinematics

	Inter-trial		Inter-session	
	
	CMC	ICC	CMC	ICC
Hallux				
Flex/Ext	0.971	0.990	0.796	0.880
Rotation	0.970	0.990	0.951	0.974
Hindfoot				
Flex/Ext	0.931	0.976	0.837	0.911
Pro/Sup	0.890	0.961	0.697	0.838
Rotation	0.927	0.974	0.728	0.820
Arch				
Height	0.959	0.992	0.798	0.883
Length	0.909	0.998	0.980	0.840
Index^*^	0.952	0.972	0.729	0.989
Forefoot				
Flex/Ext	0.978	0.986	0.840	0.913
Pro/Sup	0.993	0.968	0.687	0.814
Rotation	0.972	0.983	0.813	0.890
Medial forefoot				
Flex/Ext	0.956	0.984	0.834	0.909
Pro/Sup	0.916	0.975	0.808	0.892
Rotation	0.949	0.985	0.808	0.893
Lateral forefoot				
Flex/Ext	0.957	0.985	0.765	0.866
Pro/Sup	0.929	0.970	0.763	0.865
Rotation	0.954	0.983	0.790	0.877

We demonstrated a MFM with 15-marker set had high inter-trial and inter-session repeatability, especially in sagittal plane motion. We thought this MFM would be applicable to evaluation of the motion of the foot segment during gait.
